# Safety and Effectiveness of BPaL-Based Regimens to Treat Multidrug-Resistant TB: First Experience of an Italian Tuberculosis Referral Hospital

**DOI:** 10.3390/antibiotics14010007

**Published:** 2024-12-25

**Authors:** Gina Gualano, Maria Musso, Paola Mencarini, Silvia Mosti, Carlotta Cerva, Pietro Vittozzi, Antonio Mazzarelli, Angela Cannas, Assunta Navarra, Stefania Ianniello, Paolo Faccendini, Fabrizio Palmieri

**Affiliations:** 1 Respiratory Infectious Diseases Unit, National Institute for Infectious Diseases “Lazzaro Spallanzani” IRCCS, 00149 Rome, Italy; gina.gualano@inmi.it (G.G.); paola.mencarini@inmi.it (P.M.); silvia.mosti@inmi.it (S.M.); carlotta.cerva@inmi.it (C.C.); pietro.vittozzi@inmi.it (P.V.); fabrizio.palmieri@inmi.it (F.P.); 2 Department of Microbiology, National Institute for Infectious Diseases “Lazzaro Spallanzani” IRCCS, 00149 Rome, Italy; antonio.mazzarelli@inmi.it (A.M.); angela.cannas@inmi.it (A.C.); 3 Department of Epidemiology, National Institute for Infectious Diseases “Lazzaro Spallanzani” IRCCS, 00149 Rome, Italy; assunta.navarra@inmi.it; 4 Diagnostic Imaging Unit for Infectious Diseases, National Institute for Infectious Diseases “Lazzaro Spallanzani” IRCCS, 00149 Rome, Italy; stefania.ianniello@inmi.it; 5 Hospital Pharmacy, National Institute for Infectious Diseases “Lazzaro Spallanzani” IRCCS, 00149 Rome, Italy; paolo.faccendini@inmi.it

**Keywords:** multidrug-resistant TB, BPaL regimens, Italy

## Abstract

**Background/Objectives**: Tuberculosis (TB) is preventable and curable, but multidrug-resistant TB (MDR-TB) and extensively drug-resistant TB (XDR-TB) pose significant challenges worldwide due to the limited treatment options, lengths of therapies, and high rates of treatment failure. The management of MDR-TB has been revolutionized by all oral anti-TB drug regimens that are likely to improve adherence and treatment outcomes. These regimes include bedaquiline (B), pretomanid (P), and linezolid (L) (BPaL), and moxifloxacin if resistance to fluoroquinolones is not detected (BPaLM). Based on the evidence generated by the TB-PRACTECAL and ZeNix randomized controlled trials, BPaL/BPaLM regimens are recommended over the currently recommended longer regimens in patients with MDR-TB or monoresistance to rifampin (RR). To our knowledge, no data are currently available on the implementation of BPaL/BPaLM regimens in Italy. **Results**: Seventeen patients completed the BPaL/BPaLM regimen, with a treatment success rate of 90% (17/19), consistent with the literature data. Eleven patients out of the nineteen retained in care (58%) complained about symptoms consistent with adverse events (AEs). No treatment interruption was necessary due to AEs. **Methods**: Here, we report the real-world experience of a tertiary referral hospital for TB in Italy, from 2022 to 2024, in the management, outcomes, and adverse drug reactions of a cohort of twenty-two MDR/RR patients treated with BPaL and BPaLM regimens. **Conclusions**: BPaL-containing regimens also serve as promising options for patients with RR/MDR-TB in terms of real-life experience, but further multicentric studies are required in Europe to confirm the efficacy of shorter regimens to eliminate MDR TB.

## 1. Introduction

Tuberculosis (TB) is a preventable and usually curable disease, causing death worldwide, particularly in low- and middle-income countries. The WHO uses five categories to classify cases of drug-resistant (DR) TB: monoresistant TB; multidrug-resistant TB (MDR-TB) or rifampicin-resistant TB (RR-TB), a form of TB disease caused by a strain of *M. tuberculosis* complex resistant to rifampicin (RIF) and isoniazid (INH), or to rifampicin, respectively; extensively drug-resistant TB (XDR-TB); and pre-XDR-TB.

Pre-XDR-TB is defined as tuberculosis resistant to isoniazid, rifampicin, and the fluoroquinolones (FQs) levofloxacin and moxifloxacin. XDR-TB is TB-resistant not only to isoniazid, rifampicin, and fluoroquinolones but also to at least one of the two fundamental drugs: bedaquiline (BDQ) and linezolid (LZD) [1].

MDR-TB and XDR-TB pose significant challenges to the global efforts due to the limited treatment options, lengths of therapies, and high rates of treatment failure [2].

According to the WHO 2023 Global TB report, the estimated number of DR-TB cases in 2022 was 410,000 (95% uncertainty interval [UI]: 370,000–450,000), with an estimated 160,000 (95% UI: 98,000–220,000) deaths.

The estimated proportion of MDR/XDR TB cases in Italy was 2.8% of the total TB cases in 2017. Unfortunately, the data on those patients who started treatment are missing [3]. Globally, the DR-TB treatment success rates increased from 50% in 2012 to 60% in 2019, with 15% of MDR/RR-TB patients still dying from the disease [4].

The treatment of drug-resistant tuberculosis has recently been revolutionized by the introduction of highly effective, shorter, and less toxic oral regimens, which require shorter hospital stays (if necessary) and are generally better tolerated. These regimens are therefore expected to improve adherence and treatment outcomes [5]. In 2022, the WHO organized the revision of the guidelines for the treatment of MDR/RR-TB [6] following the completion of clinical trials evaluating new regimens with exclusively oral drugs, which included bedaquiline (B), pretomanid (Pa), and linezolid (L) (BPaL) [7].

The “TB pragmatic clinical trial for a more effective, concise and less toxic regimen” PRACTECAL (BPaL + Mfx arm 1 versus arm 2 BPaL + Cfz versus standard of care), NixTB (BPaL for 26 or 39 weeks), and ZeNix (BPaL with LZD 600 mg/die) trials evaluated the treatment outcomes of different BPaL-containing regimens [8,9,10]. Based on the evidence generated by randomized controlled trials TB-PRACTECAL [10] and ZeNix [9,10], the Guidelines Development Group (GDG) concluded that BPaL/BPaLM regimens achieve better outcomes than the longer regimens that were previously recommended in patients with MDR/RR-TB [11].

Since December 2022, pretomanid has been available for the last DR-TB patients diagnosed in the year at our institution, a tertiary regional referral center for tuberculosis in the Lazio region, Italy. From that moment on, we have been using this therapeutic regimen. We therefore want to describe how we have cared for patients, the frequency and types of adverse drug reactions we have observed, and the clinical and microbiological outcomes of the cohort of MDR/RR patients treated with the BPaL and BPaLM regimens from 2022 to 2024.

To our knowledge, in fact, no data are yet available on the implementation of the BPaL/BPaLM regimens in Italy.

## 2. Results

The characteristics of the 22 patients enrolled in the study are shown in Table 1.

The mean age of the patients at diagnosis was 42 years (range, 29–48 years). Most of the patients were male (*n* = 14 [64%]) and foreign-born (*n* = 19 [86%]) (Table 1). Seven patients (32%) reported prior treatment for tuberculosis (drug susceptibility unknown). All the patients had pulmonary localization, of whom only two (9%) had both pulmonary and extrapulmonary tuberculosis (pleural- and lymph node involvement, respectively). All had acid-fast bacilli detected on sputum smear (*n* = 22, 100%), and seventeen (77%) had cavitations on radiography.

Three patients had monoresistance to rifampin (14%), multidrug resistance was observed in fifteen (68%), and four patients (18%) had additional resistance to FQ.

Four patients out of twenty-two in which FQ resistance was detected received the BPaL regimen, while the remaining eighteen (82%) underwent the BPaLM regimen. All twenty-two patients (100%) started BPaL/BPaLM with a linezolid dosage of 600 mg daily.

Seventeen patients completed six months of treatment, being followed in our hospital (77% of the initial population), three patients were transferred (14%), and one patient was lost to follow-up during the treatment (5%). One patient (5%) died during the treatment; one other died after the end of the treatment. No culture reversion occurred within the six months prescribed by the therapeutic regimen. Seventeen patients completed the BPaL/BPaLM regimens, with a treatment success rate of 90% (17/19) (see Table 2).

Among the 22 patients with pulmonary tuberculosis who received BPaL/BPaLM regimens, the mean time to culture conversion was 37 days (range 21–67 days). The mean duration of the treatment among those who completed it was 181 days (range 180–183).

Eleven patients out of the nineteen retained in care (58%) complained about symptoms consistent with adverse events (AEs) (Table 3).

As for the neurologic AEs, two patients (11%) experienced peripheral neuropathy at the end of the treatment, with transient numbness and tingling of extremities (grade 1), but they did not require a linezolid dose or frequency adjustment. Five patients (26%) developed anemia, and no one required a blood transfusion during the linezolid treatment; the linezolid dosage was changed from 600 mg daily to 300 mg/day in one patient with grade 3 anemia, with recovery in two months.

Two patients reported nausea (grade 1) and one severe vomiting (grade 3) with the necessity to stop fluoroquinolones. The serum aspartate aminotransaminase and/or alanine aspartate aminotransaminase levels increased to >3 times the normal upper limit (40 UI/mL) in two patients (11%). In two patients, a grade 1 prolonged QTc interval was observed. The mean days of exposure until the first AE was 56 (range 27–180). No treatment interruption was necessary due to AEs. The patients were followed up for at least 6 months after the end of the treatment.

After 12 months from the treatment prescription, one additional patient died, one relapsed, and the remaining ten patients who completed one year of observation had persistently negative cultures (sustained cure). No statistically significant correlation was found between the incidence of adverse events and clinical and socio-demographic characteristics.

## 3. Discussion

In this article, we describe a cohort of 22 patients treated for RR/MDR tuberculosis with the BPaL/BPaLM regimen at an Italian tertiary referral hospital for tuberculosis in the Lazio region of Italy.

Of the twenty-two patients who started treatment, three (14%) were transferred out (one went back to Romania; two completed their treatments in another region of Italy), one patient was lost at follow-up, and one unfortunately died. Seventeen patients completed the BPaL/BPaLM regimen, with a treatment success rate of 90% (17/19), consistent with the data in the available studies [8,9,10]. The TB-PRACTECAL trial showed high treatment success rates with the BPaLM regimen (89%) of 6 months in duration compared with the previously recommended regimens (52%) [10,11].

This real-life retrospective study was conducted in Italy, a country with a low TB prevalence, in which, according to the ECDC Report, there were 39 confirmed RR-MDR TB cases in 2022 [12].

Unfortunately, no data on treatment outcomes are available in our country. However, comparing the therapeutic success rate of the BPaL-containing regimens in our hospital with the 77% previously reported, we can observe a considerable improvement in the cure rate [13]. In a recent meta-analysis including data from investigating trials, the pretomanid-containing regimen was associated with a favorable outcome 46.73 times (95% CI: 11.76–185.70) compared to the non-pretomanid regimen and BPaLM/BPaL [14]. The clinical investigative trials included participants enrolled in countries with a high DR TB burden (South Africa, Uzbekistan, Belarus, Georgia, Moldova, Russia, Tanzania, Kenya, Malaysia, etc.). To our knowledge, there are two papers describing the implementation of BPaL-containing regimes in low-prevalence settings. Traut J. and colleagues described a case series of MDR TB patients in Germany [15]. Out of the fifteen patients receiving the BPaL-containing regimens, eight patients successfully completed their 6 months of BPaL therapy, and four were still undergoing therapy. Another study conducted in the United States on a larger cohort of patients showed that, of the seventy patients starting BPaL, sixty-eight (97.1%) completed BPaL, and two of the sixty-eight (2.9%) experienced relapses after completion, with a sustained successful outcome in 97.1% of the cases [11].

We report that more than half of the patients observed during the entire treatment period of almost 6 months experienced one AE. Fortunately, no patient had to interrupt the regimen because of AEs. Systematic clinical monitoring of patients enables timely interventions regarding symptoms and adverse drug reactions, which may occur during the long months of MDR-TB treatment. Monitoring and management of AEs have been recommended by the WHO since 2006 [16].

Like other clinical aspects, adverse events and the actions taken by the patient and caregivers should be carefully recorded in the patient’s clinical documentation as these comprise an intrinsic component of good clinical practice included in the International Standards for TB Care [17] and enable patients to be safely cured.

The safety of BPaL/BPaLM regimens remains an important consideration. A recent meta-analysis by Hasan et al., demonstrated that the daily dose of 1200 mg of linezolid caused grades 3–4 adverse events in 68 of 195 (35%) randomized patients who received BPaL-containing regimens compared to 89 of 396 (22%) patients who received 600 mg of linezolid/day [18]. Although our study cohort has a much smaller sample size, we report that, with a daily dose of 600 mg of linezolid, five patients (26%) developed anemia, although no one required a blood transfusion, consistent with the previously cited study. Since it was not possible to measure the blood concentrations of the drug in our hospital, we had to reduce the linezolid dose to 300 mg/day in the patient who developed a grade 3 adverse event.

Recent data from a US retrospective cohort reported that 5.9% of those patients with neurotoxicity required a change in linezolid dose or frequency [11]. In our cohort, only two of the nineteen patients (11%) presented symptoms of peripheral neuropathy, but they were able to continue the prescribed treatment thanks to symptomatic therapy.

In our cohort, two patients experienced grade 1 QTc prolongation. In a recent paper, Motta and colleagues, in a post hoc analysis of the ECGs from a TB-PRACTECAL participant, observed a few QT-prolonging events with BPaL/BPaLM regimens, suggesting that the safe cardiac profile of BPaL-based regimens should reassure clinicians as they transition patients to new recommended regimens [19]. Our experience confirms that the benefits of the BPaL/BPaLM regimen in treating MDR-TB and XDR-TB often outweigh the risks when managed appropriately.

In clinical trials assessing the safety and efficacy of the BPaL/BPaLM regimen for the treatment of DR-TB, liver toxicity has emerged as a significant concern. In the Nix-TB trial, approximately 10% of the patients experienced elevated liver enzymes; in the ZeNix trial, liver toxicity was again documented but at similarly low rates [8]. The TB-PRACTECAL trial, which evaluated the BPaL-containing regimen, reported a slightly higher incidence of hepatotoxicity compared to earlier studies. Around 4.6% of the patients experienced significant liver enzyme elevations requiring intervention [5]. In our cohort, we could observe only two cases of liver injury, but in no case did we have to interrupt treatment. Given the hepatotoxic potential of the BPaL/BPaLM regimen, particularly pretomanid, regular liver function testing (LFT) is essential.

The guidelines recommend baseline LFT followed by periodic testing during treatment to detect any early signs of liver impairment. In cases where significant liver enzyme elevations occur, dose modifications or temporary discontinuation of the regimen may be necessary. While severe liver toxicity is rare, regular monitoring and management are essential to mitigate risks. Early detection through liver function tests can help to avoid more severe outcomes, making the regimen safer for patients with pre-existing liver conditions [6].

We found that one patient relapsed six months after he started the treatment (5%). Conceptually, the risk of MDR-TB relapse could be increased with the BPaL/BPaLM regimen, having decreased the duration of the treatment to 6–9 months. A recent systematic review and meta-analysis found an estimated relapse rate of 2.0% (95% CI, 1.0–3.0%) for all the regimens used for the treatment of MDR-TB, including BPaL [20]. Since most of the data came from trial settings, the authors conclude that studies on large programmatic cohorts with longer post-treatment follow-up periods are needed to confirm the low relapse rate currently reported.

The limitations of this analysis include passive reporting and the small number of patients enrolled. Further multicentric studies are required in Europe to explore the benefits of shorter regimens to eliminate MDR TB.

In conclusion, to ensure the best possible care for people affected by rifampicin-resistant and multidrug-resistant tuberculosis, we need to identify and develop completely oral therapeutic regimens, easily tolerated and of acceptable duration, in order to also obtain a durable cure in all patients [21]. BPaL-containing regimens offer a promising range of new options for patients with RR/MDR-TB, even if we fear the spread of resistance to FQs, bedaquiline, and pretomanid [22]. The development of these innovative therapeutic regimens will certainly contribute to achieving the ambitious goal of tuberculosis eradication in Italy.

## 4. Materials and Methods

The data of the 22 patients affected by RR/MDR-TB, monitored from December 2022 to June 2024 at the National Institute for Infectious Diseases—INMI “L. Spallanzani” in Rome, were collected from the clinical documentation and anonymized. Patient characteristics regarding diagnosis were defined according to WHO definitions [13].

The laboratory diagnostic of tuberculosis workflow on primary samples included smear microscopy and molecular assays. Liquid cultures were carried out for rapid automated cultivation with Mycobacteria Growth Indicator Tubes (MGITs), incubated in a dedicated instrument (MGIT 960; Becton Dickinson, Franklin Lakes, NJ, USA).

Molecular assays on primary samples are Xpert MTB/RIF (Cepheid, Sunnyvale, CA, USA), GenoType MTBDR plus/sl (Hain Lifescience GmbH, Nehren, Germany), AnyplexTM II MTB/MDR, MTB/XDR Detection (Seegene, Seoul, Republic of Korea), and BD MAX™ MDR-TB, which are performed on a selection of samples in order to accelerate therapy implementation. Following isolation of *M. tuberculosis*, phenotypic (pDST) and genomic (gDST) drug sensitivity testing were performed. As described by Cannas A. et al. [23]: “Automated pDST for first-line drugs was performed using the MGIT automated system (critical concentrations: RIF 1.0 or 0.5 mg/L, following WHO recommendations at the time of testing INH 0.1 and 0.4 mg/L; EMB 5.0 and PZA 100 mg/L). The pDST for second-line drugs [moxifloxacin (MFX), levofloxacin (LFX), amikacin (AMK), BDQ, clofazimine (CFZ), cycloserine (DCS) and LZD] was performed and analyzed using a semi-automated system (BD EpiCenter™ TB-eXiST, Becton, Dickinson and Company, Franklin Lakes, NJ, USA). Whole-genomic sequencing (WGS) was performed on genomic DNA extracted from all culture isolates included in this study. Sequencing was carried out using the Illumina systems (Illumina Inc., San Diego, CA, USA). Briefly, 150 bp or 300 bp paired reads were generated using Nextera XT DNA Library Prep Kit and MiSeq sequencers (Illumina Inc., San Diego, CA, USA). The reference genome breadth was ≥92% with a mean depth of coverage of ≥30×. Sequence reads obtained with WGS were submitted to PhyResSE v1.0 pipeline12 in order to predict susceptibility or resistance based on identification of mutations associated with drug resistance. Mutations identified in the genes rpoB, katG, and inhA-promoter were further characterized in MDR-TB strains, and frequencies were calculated”.

Patients were treated according to the institutional protocol, drawn up following WHO MDR-TB guidelines [24]. Treatment included bedaquiline 400 mg daily for 14 days, then 200 mg thrice weekly (TIW), pretomanid 200 mg daily, and linezolid 600 mg/daily (BPaLM regimen), with directly observed therapy. Moxifloxacin 400 mg/daily was added if resistance to FQ not detected (BPaLM regimen).

Before treatment, patients underwent history, physical examination, laboratory testing (hemogram, blood biochemistry including magnesium, metabolic and liver panel, thyroid function, human immunodeficiency virus [HIV] serology, and pregnancy test), CT scan and/or chest radiography, electrocardiography, and visual acuity testing.

Patients began treatment as inpatients to ensure that AFB sputum conversion was achieved on three negative samples and to monitor drug tolerance. Clinical laboratory tests and ECG were repeated monthly in the outpatient clinic, or more frequently if necessary, until treatment was completed. Mycobacterial culture and direct sputum smear examination were performed monthly.

All patients were thoroughly informed about the diagnosis of MDR-TB, treatment plan, and potential adverse events, and the importance of reporting any adverse events to their physician when they occurred was emphasized. During each follow-up visit, patients were able to report any onset of symptoms and adherence to treatment. Demographic and clinical data (age, sex, medical history, and treatment regimen) and adverse events were recorded by a researcher from the group with clinical experience in TB treatment and data management. MDR-TB treatment outcome was defined according to the WHO definition [13].

Adverse drug reactions were classified according to WHO definition in adverse events (AEs) and serious adverse events (SAEs) [13]. Adverse events were managed according to WHO guidelines and our institutional protocol [24]. All SAEs and AEs have been graded for severity according to the Severity Grading Scale (grades 1–5) [14].

The study was approved by the Ethics Committee of the National Institute for Infectious Diseases “L: Spallanzani”—IRCCS with decision no. 12 (17 February 2015). All patients included in this study provided their written consent to participate and signed the authorization to use anonymized clinical data.

## Figures and Tables

**Table 1:** Baseline patient characteristics (*N* = 22).

Characteristic		Patients, No. (%)
	Age, mean (range), years	42 (range 29–48)
	Male sex	14/22 (64%)
	Born outside Italy	19 (86%)
Baseline comorbid conditions		
	HIV infection	none
	HCV Infection (concomitant treatment with sofosbuvir/velpatasvir)	2 (9%)
	Diabetes	2 (9%)
	Renal disease	none
	Liver disease or alcohol abuse	4 (18%)
	Cognitive impairment	2 (9%)
	Concomitant drug prolonging QTc	1 (5%; quetiapine)
Tuberculosis disease characteristics		
	New TB cases	15 (68%)
	RR-TB	3 (14%)
	MDR-TB	15 (68%)
	Pre-XDR-TB	4 (18%)
	Pulmonary	22 (100%)
	Extrapulmonary only	none
	Both pulmonary and extrapulmonary sites	2 (9%) Pleural Lymphnodes
	Cavitation on chest radiograph	17 (77%)
	Positive sputum AFB smear	22 (100%)
	Positive molecular test	22 (100%)
	Positive mycobacterial culture	22 (100%)
	Other positive culture, any site	none
	Bilateral involvement	12 (55%)

**Table 2:** Treatment outcomes (according to WHO definitions).

Type of Outcome	Outcomes at the End of 6 Months Treatment Patients N. (%)	Outcomes 6 Months After the End of Treatment Patients N. (%)
Cured	17/19 (90%) (excluded transferred patients)	16/19 (84%) (sustained treatment success)
Transferred out	3/22 (14%)	3/22 (14%)
Failed	0	1/19 (5%)
Lost to follow-up	1/19 (5%)	1/19 (5%)
Died	1/19 (5%)	2/19 (10%)

**Table 3:** Serious adverse events (SAEs) and adverse events (AEs) reported among patients retained in care.

AE	AE Patient No. (WHO Grade)	Action Required	SAE Patients (Grade)	Median Days of Exposure Before SAE	Action Required
Gastrointestinal intolerance (nausea, vomiting, diarrhea, dispepsia)	2 (Grade 2)	Supportive treatment	1 (Grade 3)	120	Interruption of offending drug (fluoroquinolone)
Liver toxicity (AST/ALT elevation)	2 (Grade 1)	Counselling on diet	0	-	-
Anemia	4 (Grade 1)	Supportive treatment (iron supplementation)	1 (Grade 3)	36	Reduced dosage of Linezolid (300 mg/day)
Peripheral neuropathy	2 (Grade 1)	Supportive treatment (pregabalin)	0	-	-
Cardiac abnormalities (QTc prolongation)	2 (Grade 1)	Monitoring	0	-	-

Legend: SAEs and AEs have been graded for severity according to the Severity Grading Scale (grades 1–3) as follows: Grade 1: Mild; asymptomatic or mild symptoms; clinical or diagnostic observations only; intervention not indicated. Grade 2: Moderate; minimal, local, or noninvasive intervention indicated; limiting age-appropriate instrumental Activities of Daily Living (ADLs). Grade 3: Severe or medically significant but not immediately life-threatening; hospitalization or prolongation of hospitalization indicated; disabling; limiting self-care ADLs. (cit. Common Terminology Criteria for Adverse Events (CTCAE) v5.0, Publish Date: 27 November 2017).

## Data Availability

All relevant data are within the manuscript. Raw data are accessible, if requested, from National Institute for Infectious Diseases “L. Spallanzani” Library to this e-mail address: biblioteca@inmi.it.
